# Novel platelet-rich plasma/ hyaluronic acid lyophilized formulations for wound healing applications

**DOI:** 10.3389/fbioe.2025.1619633

**Published:** 2025-09-10

**Authors:** Antonella D’Agostino, Maria d’Agostino, Marta Nardini, Anita Muraglia, Celeste Di Meo, Maddalena Mastrogiacomo, Chiara Schiraldi

**Affiliations:** ^1^ Department of Experimental Medicine, Section of Biotechnology Medical Histology and Molecular Biology, University of Campania “Luigi Vanvitelli”, Naples, Italy; ^2^ Biotherapies Laboratory, Department of Internal Medicine and Medical Specialities, University of Genoa, Genoa, Italy

**Keywords:** hyaluronic acid, platelet-rich plasma, wound healing, *in vitro* scratch assays, proliferation, size exclusion chromatography–triple detector array, time-lapse video microscopy, biomarkers

## Abstract

Platelet-rich plasma (PRP) is a well-known biological product used in regenerative medicine. One of the limitations of its clinical use is the need for surgeons to obtain ready-to-use preparations. A lyophilized formulation with specific and reproducible levels of growth factors can, therefore, be considered a significant improvement for tissue regeneration. Therefore, it is of great interest to develop a formulation that enables the prolonged release of the growth factors and bioactive components present in PRP while improving the stability of these biomolecules during storage. To this aim, specific preparations obtained by mixing hyaluronic acid (HA) of low–medium molecular weight (56 or 200 kDa) with PRP were lyophilized to achieve an “off-the-shelf” product. These formulations were characterized from both biophysical and biological perspectives. Primary human dermal fibroblast proliferation and time-lapse scratch assays were performed on freshly lyophilized formulations and during storage at different temperatures (4°C and 25°C) to assess their biological activity upon resuspension for up to 6 months. Gene and protein expressions of collagen type 1 and elastin at time zero were evaluated. Platelet-Derived Growth Factor (PDGF)-BB and Vascular Endothelial Growth Factor (VEGF) releases were measured using the ELISA assay. All HA/PRP formulations were able to induce cell proliferation compared to PRP alone. HA/PRP formulations exhibited a superior repair rate compared to PRP in the scratch assay, confirming HA’s ability to improve fibroblast migration. In the presence of HA, 80% of wound closure was achieved within 24 h, whereas PRP-treated samples reached approximately 60% of the repaired area. These data were supported by collagen and elastin expression levels. In *in vitro* wound healing assays, lyophilized HA/PRP products exhibited a superior effect compared to PRP alone at 3 months, but no significant improvement was found after 6 months. Prolonged storage needs very low temperatures to preserve PRP features (−20°C). In conclusion, we propose novel lyophilized HA/PRP formulations as promising products for topical and intra-dermic applications, especially for wound healing. The combination of HA as a biopolymer facilitates the slow release of growth factors contained in PRP while also allowing for a prolonged shelf life under cold-controlled conditions (4°C).

## 1 Introduction

Novel approaches and formulations based on Platelet-Rich Plasma (PRP), derived using different technologies, are continuously being proposed for diverse treatments in the regenerative medicine field. Scientific and clinical studies support the functionality of PRP in treating various conditions, such as osteoarthritis/musculoskeletal diseases, soft tissue injuries, and wound healing. Among the many valuable beneficial effects of PRP, a major effect was assessed in its ability to improve dermal tissue repair, as reported for *in vitro* and *in vivo* studies, also reducing the risk of infection (Cao et al., 2021; Kirsner and Eaglstein, 1993).

Inflammation is the most crucial phase of wound healing, characterized by the biosynthesis and recruitment of pro-inflammatory cytokines. This phase is preceded by growth factor release, promoting epithelialization and angiogenesis during the proliferative phase. In particular, chronic wounds are associated with reduced growth factor levels in the wound bed, along with persistent inflammatory markers and overexpression of remodeling proteins, which hinder the healing process (Han and Ceilley, 2017). Recently, de Castro et al. investigated the use of PRP as a therapeutic approach for stretch marks, highlighting its ability to modulate inflammatory cytokines, remodel the extracellular matrix (ECM) and improve tissue texture and quality (de Castro Roston et al., 2023). When PRP is activated, a gel is formed, enabling positive clinical outcomes in wound healing as a therapeutic approach (Nguyen and Pham, 2018; Lacci and Dardik, 2010; Carter et al., 2011). Although the literature reports beneficial effects of PRP, some meta-analyses of clinical trials have shown that, in chronic wounds of elderly patients, the use of autologous PRP alone is often ineffective (Martinez-Zapata et al., 2016; Qu et al., 2021; Li et al., 2020; Zhao et al., 2020).

The direct application of PRP in gel form to wounds presents several challenges, including poor mechanical compatibility with native skin, limited adhesion to the wound site (Rodriguez et al., 2014), and a rapid, uncontrolled release of growth factors, which may reduce its sustained therapeutic effectiveness.

A controlled and prolonged release of these bioactive molecules into the wound bed is, therefore, highly desirable, leading to increased interest in the development of biomaterial- or biopolymer-based formulations (Xie et al., 2014). Although there is limited information on allogeneic applications of PRP and further research is needed, variability exists even within autologous PRP preparations, depending on the individual’s health status (Akbarzadeh et al., 2021).

Another critical aspect is the ability of activated platelets within the gel to release growth factors over time at effective doses (Spanò et al., 2018). A recent literature study describes a system of cross-linked chitosan and gelatin loaded with PRP, in which appropriate photostimulation induces PRP activation, thus enabling controlled growth factor release better than conventional methods (Koyuncu et al., 2022). Allogeneic PRP containing standardized levels of growth factors, such as Platelet-Derived Growth Factor (PDGF), Vascular Endothelial Growth Factor (VEGF), and Fibroblast Growth Factor (FGF), has been recently proposed in combination with biopolymers to enhance processability, adhesiveness and spreading on the wound, with a slow and sustained release of these bioactive molecules (Werner and Grose, 2003; Barrientos et al., 2008; Werner and Grose, 2003). Hyaluronic acid (HA), with well-established features, has already been used in combination with PRP to create a gel that entraps growth factors, allowing slow delivery and possibly prolonging their stability (Rao et al., 2022). However, Rao et al. used high-MW HA (HHA), widely exploited in tissue repair (D’Agostino et al., 2019). Research on the correlation between HA size and biological function suggests that specific HA fractions (ranging from 50 to 500 kDa) are optimal for skin repair (D'Agostino et al., 2017). For this reason, low/medium-MW HAs were considered the most suitable biopolymers to be used as carriers to entrap “active principles.” These highly biocompatible HAs exhibit lower viscosity than HHA; thus, they can be more easily handled at higher concentrations than HHA. The aim of this study was to design novel freeze-dried formulations that provide a sustained release of the growth factors and active compounds contained in PRP while potentially enhancing their stability and cryopreservation. Novel formulations based on pharma-grade HA families, resembling those already characterized in previous studies (MW = 56 ± 2 kDa and 200 ± 10 kDa) (Di Meo et al., 2024), with full hydrodynamic characterization and PRP, were obtained through mixing and lyophilization, resulting in a dried powder suitable for storage and easy reconstitution upon the addition of highly purified water for the intended applications. The stability of HA/PRP preparations during storage was investigated at 4°C and 25°C, temperatures considered relevant for the intended biomedical applications. These new formulations, beyond incorporating PRP, showed interesting bio-reparative properties by coupling the beneficial effects of HA as a supporting biopolymer with those of PRP upon reconstitution and use, even after 6 months of storage.

## 2 Materials and methods

### 2.1 Materials

Pharma-grade hyaluronic acid samples of different molecular weights (56 and 200 kDa) were available in our laboratory, kindly provided either by Altergon Italia Srl or by IBSA Farmaceutici Italia Srl (Italy) within ongoing projects (Invitalia MISE 000463 and MIMIT CrossGAG CUP: B29J24001220005).

### 2.2 Methods

#### 2.2.1 PRP preparation

PRP was derived from expired buffy coat (BC) samples obtained from the whole blood of healthy donors from the Transfusional Centre of the IRCCS Ospedale Policlinico San Martino of Genova. Blood was collected using citrate phosphate dextrose as anticoagulant. All the procedures described were performed in a closed bag system. Ten buffy coat samples were pooled together and centrifuged at 388 *rcf* for 10 min at 22°C, resulting in the separation of PRP in the upper phase and red blood cells in the lower phase. The PRP was recovered and centrifuged at 2,169 *rcf* for 20 min at 22°C to sediment the platelets, and the upper phase, represented by platelet-poor plasma, was recovered and used to adjust the platelet concentration to 2.5 × 10^6^ platelets/μL. PRP was then frozen and stored at −20°C until further processing. The decision to use this platelet concentration was based on previous results obtained by our group from an experimental study on the development of a PRP-based membrane to be used as a wound-healing inducer in a chronic ulcer animal model (Spanò et al., 2018). Recent literature suggests that cryopreserving PRP at −20°C (or −80°C) for up to 12 months is a feasible approach for retaining its therapeutic potential. In particular, storage at −20°C allows PRP to maintain a platelet concentration similar to that at −80°C, a stable concentration of growth factors, and efficient biological activity of the frozen product (Beitia et al., 2025). The PRP preparation had a WBC count of approximately 0.2 × 10^3^/μl, which is generally considered a low WBC contamination and is supposed to minimally modulate the *in vitro* PRP regenerative effect (Giusti et al., 2018).

#### 2.2.2 Hydrodynamic characterization of HA samples

The hydrodynamic characterization of hyaluronan powders, appropriately resuspended and solubilized in purified water, was performed via size exclusion chromatography–triple detector array (SEC–TDA) using Viscotek (TDA 305, Malvern, United Kingdom). The measuring system and the analytical protocol were described in detail by Di Meo et al. (2024). In brief, 200 μL of HA solution at 20 mg/mL was withdrawn before the sterilization protocol, diluted in distilled water to 1.5 mg/mL, filtered through a 0.22-µm filter, and analyzed by SEC–TDA at 40°C with a chromatographic run of 1 h. The hydrodynamic measurements, including weight and numeric average molar mass (M_w_ and M_n_, respectively), polydispersity index (M_w_/M_n_), intrinsic viscosity ([η]), and hydrodynamic radius (R_h_) distributions, were provided. Analyses were run at least in triplicate for each sample.

#### 2.2.3 Preparation of HA/PRP samples

An appropriate amount of HA was dissolved in ultrapure water to reach a final concentration of 20 mg/mL, and it was left under mild shaking at room temperature and 400 rpm on a Multi Reax Heidolph Mixer (Heidolph Instruments, Schwabach, Germany). The solutions were then sterilized in an autoclave (Autoclave, Vapor Matic 770, Italy; F_0_ = 12 min at 121°C, 1 bar). PRP was thawed at 37°C and directly added in a 1:1 ratio to the hyaluronan solutions. The HA/PRP mixtures were left stirring for approximately 1 h at room temperature and then aliquoted into glass vials and frozen at −20°C. PRP alone was also frozen and used as a control.

The frozen samples were freeze-dried overnight (Heto LyoPro 3000) and then stored at controlled temperatures of 4°C and 25°C for 3 and 6 months before their use. The resulting samples were briefly labeled HA56/PRP, HA200/PRP, and PRP, depending on the presence or absence of the biopolymer with different molecular weights.

#### 2.2.4 Characterization of PRP/HA formulations

##### 2.2.4.1 Rheological analysis

Samples were prepared by dissolving the lyophilized powders in 1 mL of highly purified water. To obtain quite homogeneous dispersion/suspension, several extrusions through a 27-gauge needle were needed.

Rheological characterization of the formulations was performed using a Physica MCR 301 oscillatory rheometer (Anton Paar, Germany), with a cone-plate CP 50–2 geometry (cone diameter 49.970 mm, cone angle 1.995°, and truncation 207 µm) and a Peltier system for temperature regulation.

Analyses were run at 37°C, and before performing the measurement, the samples were left to equilibrate for 5 min for leveling the temperature distribution. Each formula was analyzed at least in triplicate under the same experimental conditions to verify the reproducibility of measurements.

A preventive amplitude sweep test was carried out over a strain range of 0.1%–100% at a constant frequency of 1.59 Hz to determine the range of linear viscoelasticity (LVE), in which the loss and the storage moduli remain constant with amplitude. Subsequently, frequency sweep tests were performed at 1% of strain (value selected within LVE range), and the profiles of the elastic modulus (G′), viscous modulus (G″), tan delta (G’’/G′), and complex viscosity (η *) were derived as a function of frequency in the range 0.159–15.9 Hz, in no-time-setting mode, with 10 measuring points/decade.

##### 2.2.4.2 Morphological analyses by SEM

Lyophilized samples (PRP, HA56/PRP and HA200/PRP) were observed via scanning electron microscopy (SEM) at different magnifications. In brief, the samples were mounted on a stub and coated with gold (Denton Vacuum Desk V) before observation with SEM (Supra 40 ZEISS; EHT = 5.00 kV, WD = 22 mm, detector in lens) (De Rossi et al., 2012).

##### 2.2.4.3 Total protein and growth factor release

*In vitro* release tests were performed in 24-well plates. The PRP or HA/PRP gel was prepared by gently mixing, in the well, 190 µL PRP or HA/PRP formulation (after prior reconstitution in sterile water) with 10 µL of 0.1 g/mL CaCl_2_. The polymerization reaction occurred after approximately 30–45 min at 37°C. After PRP gelation, 0.3 mL of PBS was added to each well containing the gel. Gels were maintained in an incubator at 37°C and 5% CO_2_. The solution containing the released proteins was harvested at 2, 4, 24, 48, 120, and 168 h from gel preparation, and after the platelet release recovery, the same starting volume of fresh PBS was replaced. Approximately 0.3 mL of conditioned medium was recovered at each time point, aliquoted, and stored at −20°C for further analysis. The total protein content in the recovered samples was determined using the Bradford assay, and the amounts of PDGF-BB (Human PDGF-BB ELISA Kit, RayBiotech Inc., United States of America) and VEGF (Human VEGF Elisa Kit, Novex, Life Technologies, United States of America) were evaluated using the ELISA assay, according to the manufacturer’s instructions. All measurements were performed in duplicate.

##### 2.2.4.4 Primary cell isolation and expansion

Human skin fibroblasts (hFBs) were obtained from discarded skin fragments from patients undergoing reconstructive mastoplastic surgery after the informed consent signature. The skin was separated from the fat layer, and the extracted pieces were disinfected by immersion in 70% ethanol, followed by two washes in sterile 1× PBS. The superficial skin was then cut using scalpels into ∼1 mm pieces that were transferred to a 10 cm Ø Petri dish. A sterile slide was placed over the skin fragments to promote the sprouting of cells. Culture was performed in α-MEM supplemented with 10% FBS, 100 UI/mL penicillin, 100 μg/mL streptomycin, and 2 mM L-glutamine in a humidified atmosphere (95% air and 5% CO_2_, v/v) at 37°C. Fibroblasts started to migrate out of the fragments within 7 days. At confluence, fibroblasts were detached using 0.05% trypsin–0.01% EDTA, counted, and frozen in several aliquots at passage 0 to use the same cells at different time points.

##### 2.2.4.5 Cell proliferation assay

At 0, 3, and 6 months from preparation and storage at the different temperatures, the effects of lyophilized HA/PRP preparations on cell proliferation were evaluated using the colorimetric CCK-8 assay (Dojindo Laboratories, Japan). The freeze-dried preparations were reconstituted in 1 mL of sterile water (to achieve the starting platelet concentration for each preparation). A total of 800 dermal fibroblasts/well at passage 2 were plated in six replicas in a 96-well plate in 100 µL α-MEM supplemented with the reconstituted products represented by 5% v/v PRP alone, 5% v/v HA56/PRP, or 5% v/v HA200/PRP in the presence of 5 UI/mL sodium heparin (Pfizer, Italy) to prevent gel formation. FCS 10% was used as a standard medium supplement for control cell cultures (CTR). Cell viability, as a proliferation index, was tested the following day (t_0_) and then after 24, 48, 72, 144, and 168 h from plating. In brief, at each experimental time point, 10 µL of the CCK-8 solution was added to 100 µL of medium/well, followed by incubation for 3 h at 37°C in the dark. Empty wells with serum-free α-MEM were used as the negative control for staining. Absorbance was measured at 450 nm using a microplate reader. Considering the complexity of the biological assay and the need to test the different stored products, the complete experiment was repeated using two different primary cultures of skin fibroblasts.

##### 2.2.4.6 *In vitro* fibroblast scratch test using time-lapse video microscopy

Lyophilized PRP, HA56/PRP, and HA200/PRP were reconstituted in 1 mL of sterile water. The obtained samples were diluted in a 1:20 ratio and then 1:5 in the medium without FCS to maintain a PRP concentration equivalent to that of the control sample (FCS 1%). A total of 50 × 10^3^ hFB cells (passage 1) were seeded in two 12-well plates (to conduct the experiment in duplicate) until complete confluence was reached. Successively, the confluent cell monolayer in each well was scratched with a sterile tip (Ø = 0.1 mm); the debris was washed with sterile PBS, and the HA/PRP formulations, diluted as mentioned above, were added. In particular, the samples at time zero and those stored at 4°C and 25°C for 3 and 6 months were tested. The *in vitro* cell migration was followed by time-lapse video microscopy (TLVM) experiments (Okolab, Italy), as described by D’Agostino et al. (2019). In brief, TLVM was assembled using an inverted microscope (AxioVision200, Zeiss Axiovert 200, Germany), a CCD-gray-camera (ORCA ER, Hamamatsu Photonics, Japan), and a motorized stage incubator that logged the position and maintained the *in vitro* cell culture conditions (37°C and 5% CO_2_ in humidified air); custom-tailored OKO-Vision 4.3 software follows the overall process and allows the image analysis. TLVM allows real-time monitoring of wound repair by recording representative images of the scratched area selected at the beginning of the experiments. The quantitative analysis of the wound closure rate was calculated as a reduction in the area over time A(t) normalized to the area measured at time 0 A (t_0_). For each well, a minimum of five fields of view were selected to derive the averaged curves of wound closure as a function of time.

##### 2.2.4.7 qRT-PCR analyses and remodeling biomarkers

Remodeling biomarkers were evaluated after 6 h of PRP, HA56/PRP, and HA200/PRP treatments on scratched monolayers. Cells were directly lysed with TRIzol^®^ (Invitrogen, Italy), and total RNA was extracted from hFB through precipitation with isopropyl alcohol. After washing with 75% ethanol, the RNA pellets were re-suspended in nuclease-free water, and RNA concentration was determined using a NanoDrop spectrophotometer (Celbio, Italy). Then, 0.5 μg of DNase-digested total RNA was retro-transcribed into cDNA using the Reverse Transcription System Kit (Promega, Italy). Quantitative real-time PCR (qRT-PCR) was obtained using iQ™ SYBR^®^ Green Supermix (Bio-Rad Laboratories Srl) to analyze Collagen type I (*COL1A1*, TIB Molbiol, Italy) and Elastin (*ELS*, TIB Molbiol, Italy) gene expressions. The primer sequences (Table 1) were used at the final melting curve from 57°C to 60°C, for *COL1A1* and *ELS*, respectively. Evaluation of genes expression was calculated using the 2^−ΔΔCT^ comparative threshold method (ΔΔCt = difference in ΔCt between treated and non-treated cells used as controls). Samples were run in duplicate, and the expression of specific mRNA was reported as normalized fold increase, calculated by the ratio of the crossing points of the amplification curves of the target genes relative to hypoxanthine phosphoribosyl transferase (HPRT, TIB Molbiol, Italy), used as the internal standard, using Bio-Rad iQ™5 software (Bio-Rad Laboratories Srl).

**TABLE 1:** Primer sequences for qRT-PCR.

Gene name	Primer sequence (5’–3′)	At PCR
HPRT	FORWARD 5′- CATCCTGCACCACCAACTG-3′ REVERSE 5′- CACAGTCTTCTGAGTGGCAG -3′	55°C
COL1A1	FORWARD 5′- CCAGAAGAACTGGTACATCA -3′ REVERSE 5′- CCGCCATACTCGAACTGGAA -3′	57°C
ELS	FORWARD 5′-AGGTGTATACCCAGGTGGCGTGCT -3′ REVERSE 5′- CAACCCCTGTCCCTGTTGGGTAAC -3′	60°C

##### 2.2.4.8 Western blotting analyses on cell extracts from wound healing experiments

Western blot analyses were performed after 24 h of treatment with PRP, HA56/PRP, and HA200/PRP samples. Cells were lysed with the RIPA buffer, and protein concentration was determined using the Bradford method. Intracellular proteins were loaded and resolved using 10% SDS–PAGE. The proteins, once separated, were transferred to the nitrocellulose membrane (Amersham). The membrane was then blocked in 5% milk dissolved in Tris-buffered saline and 0.05% Tween-20. Anti COL1A1 (110 kDa, Elabscience, Houston, TX, United States) and ELS (60 kDa, Elabscience) primary antibodies were used at 1:250 dilutions. Immunoreactive bands were detected by chemiluminescence using the corresponding horseradish peroxidase-conjugated secondary antibody (Santa Cruz Biotechnology (Santa Cruz, CA); 1:3000 dilutions) and developed using an ECL system (Chemicon-Millipore, United States). Protein levels were normalized to the signal of anti-glyceraldehyde-3-phosphate dehydrogenase polyclonal antibody used as housekeeping protein (GAPDH, Elabscience; 1:1000 dilutions). The semi-quantitative analysis of protein levels was carried out using the Gel Doc 2000 UV System and the Gel Doc EZ Imager (Quantity One software, Bio-Rad Laboratories, Italy).

##### 2.2.4.9 Statistical analyses

Statistical studies were performed using the t-test or ANOVA test. In particular, cell proliferation/total protein-growth factor release and wound healing assay results were analyzed with ANOVA and Tukey *post hoc* correction using GraphPad Prism 9.4.0 and JASP software (JASP 0.13.0.0, Netherlands), respectively. qRT-PCR and Western blotting results were compared using Student’s t*-test. p-*values <0.05, 0.01, 0.001, or 0.0001 were considered for significant differences and specifically indicated in figures with *, **, ***, and **** symbols, respectively.

## 3 Results

### 3.1 Hydrodynamic characterization of hyaluronic acid

Hydrodynamic parameters of the HAs used in this research activity are reported in Table 2. SEC–TDA results showed that the average molecular weights of the samples are 56 kDa and 200 kDa, respectively, and the molecular weight distributions observed for the samples (M_w_/M_n_≃1.7) confirmed the high purity of the starting powders, which is quite narrow for low-MW hyaluronan populations. The intrinsic viscosity and hydrodynamic radius obtained for each sample were in line with the values found in the literature for unmodified hyaluronic acids of comparable molecular weight.

**TABLE 2:** SEC–TDA results for the HA raw materials. Analyses were run in triplicate.

Sample	M_w_ (kDa)	M_n_ (kDa)	M_w_/M_n_	[η] (dL/g)	R_h_ (nm)
HA56	56 ± 2	30 ± 5	1.7 ± 0.2	1.6 ± 0.1	10.9 ± 0.2
HA200	200 ± 10	130 ± 30	1.7 ± 0.3	5.6 ± 1	25.2 ± 0.5

Sample molecular weight (weight average molar mass, M_w_; numeric average molar mass, M_n_; polydispersity index, M_w_/M_n_), intrinsic viscosity ([η]), and molecular size (hydrodynamic radius, R_h_) are calculated as the mean ± standard deviation.

### 3.2 Rheological evaluation of PRP-based formulations

**FIGURE 1:**
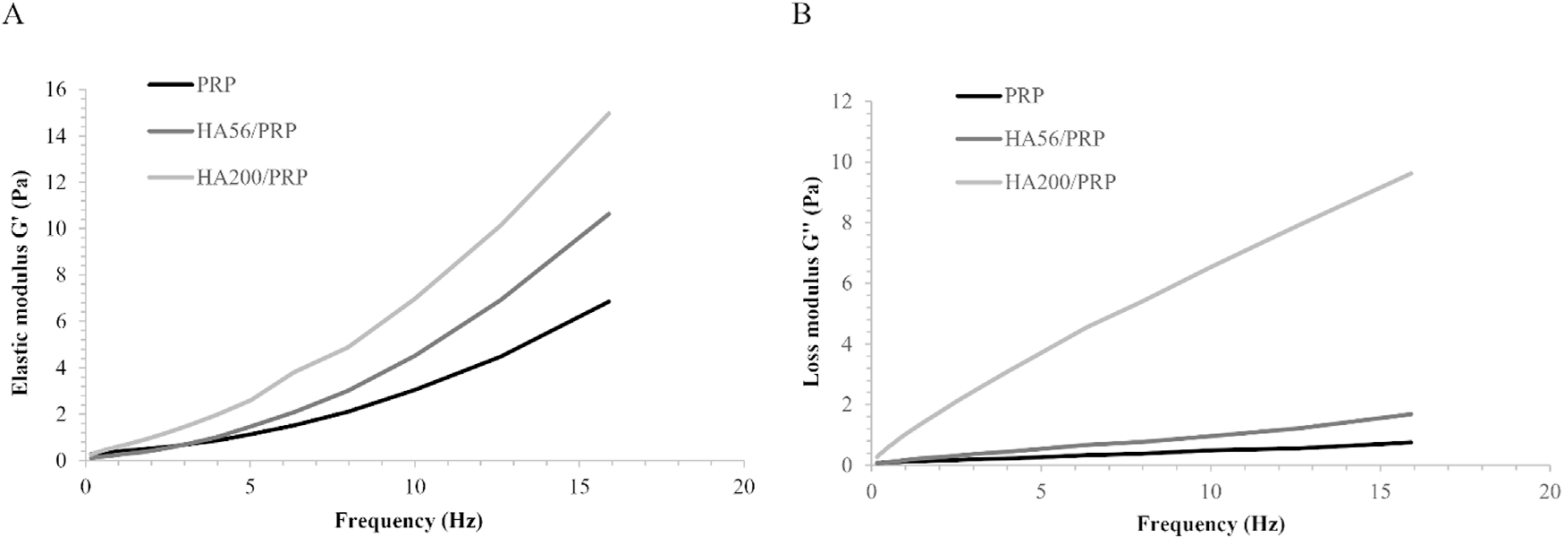
Sample frequency sweep test at 37°C in the range of frequency 0.159–15.9 Hz: **(A)** elastic modulus G’; **(B)** viscous modulus G’’.

Results of samples’ rheological evaluation are reported in Figure 1. The mechanical spectra showed that both viscoelastic moduli increased with frequency over the entire range tested for all preparations. Statistical data acquired using a t-test at each frequency point revealed that both G′ and G″ of HA200/PRP were significantly higher than those of HA56/PRP and single PRP throughout the entire frequency range. In contrast, G′ and G″ of HA56/PRP are significantly different from those of PRP only at frequencies above 10 Hz. These findings indicate that the presence of 56 kDa HA has a limited impact on the rheological behavior of the PRP-based formulation, particularly at lower frequencies. The overlay of the elastic and viscous modulus profiles of HA200/PRP revealed the typical viscoelastic behavior of unmodified hyaluronan solutions, with a crossover point of approximately 12.6 Hz. No crossover points were detected for the other samples.

### 3.3 Morphological characterization by SEM

**FIGURE 2:**
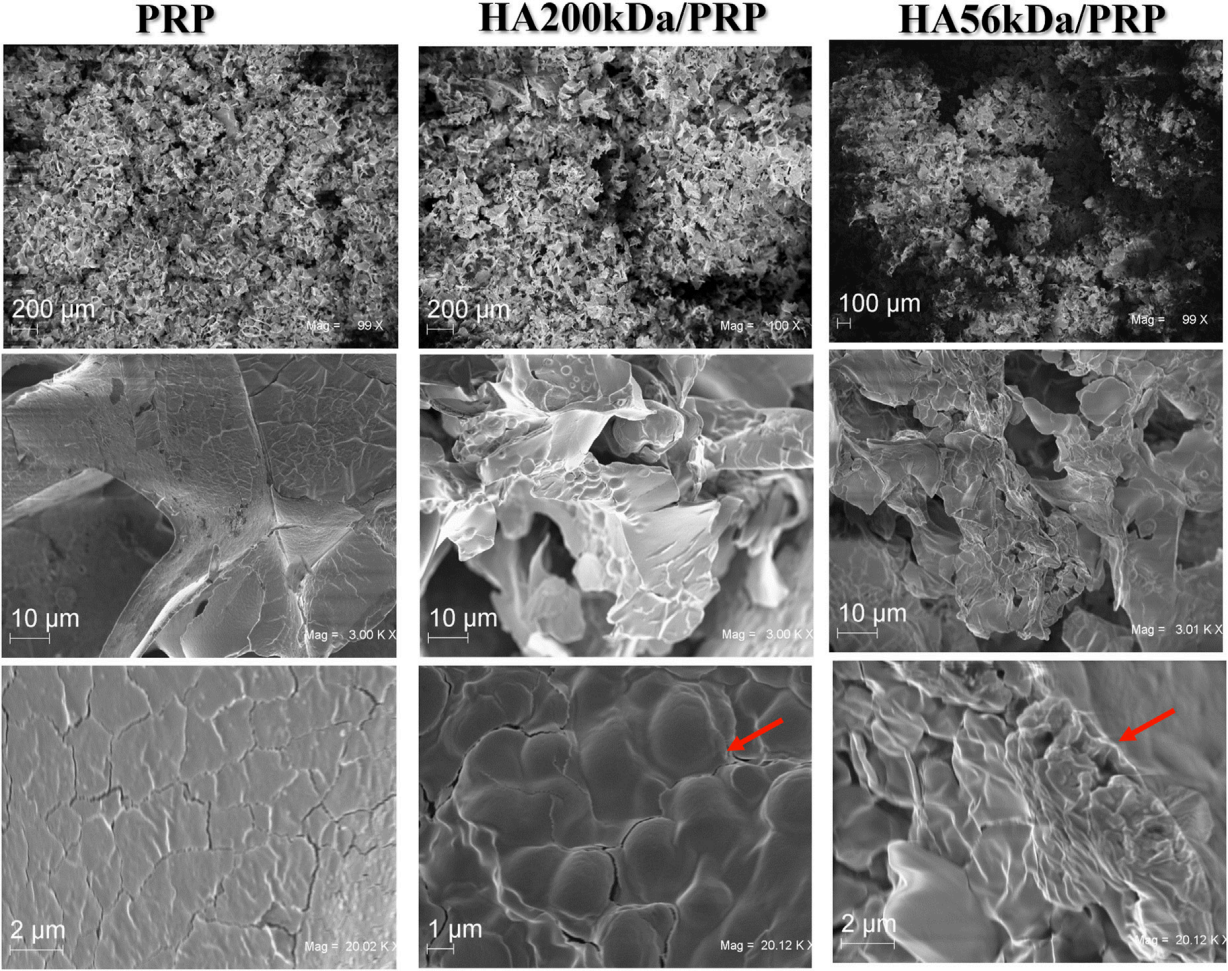
SEM of surface morphology of HA200/PRP and HA56/PRP compared to PRP alone at different magnification (first row ∼×100; second row ∼×3,000; last line∼×20,000).

SEM observations were performed on lyophilized samples and revealed structures slightly different depending on the formula. PRP images, as expected (Figure 2), were very similar to those reported in the literature (Qi et al., 2015). At higher magnification (3.00 KX), we observed that the addition of HA to PRP resulted in more compact formulations, almost resembling a continuous gel. It appeared that HA entrapped PRP particles in a structure resembling granules (red arrows).

### 3.4 Total protein and growth factor release

**FIGURE 3:**
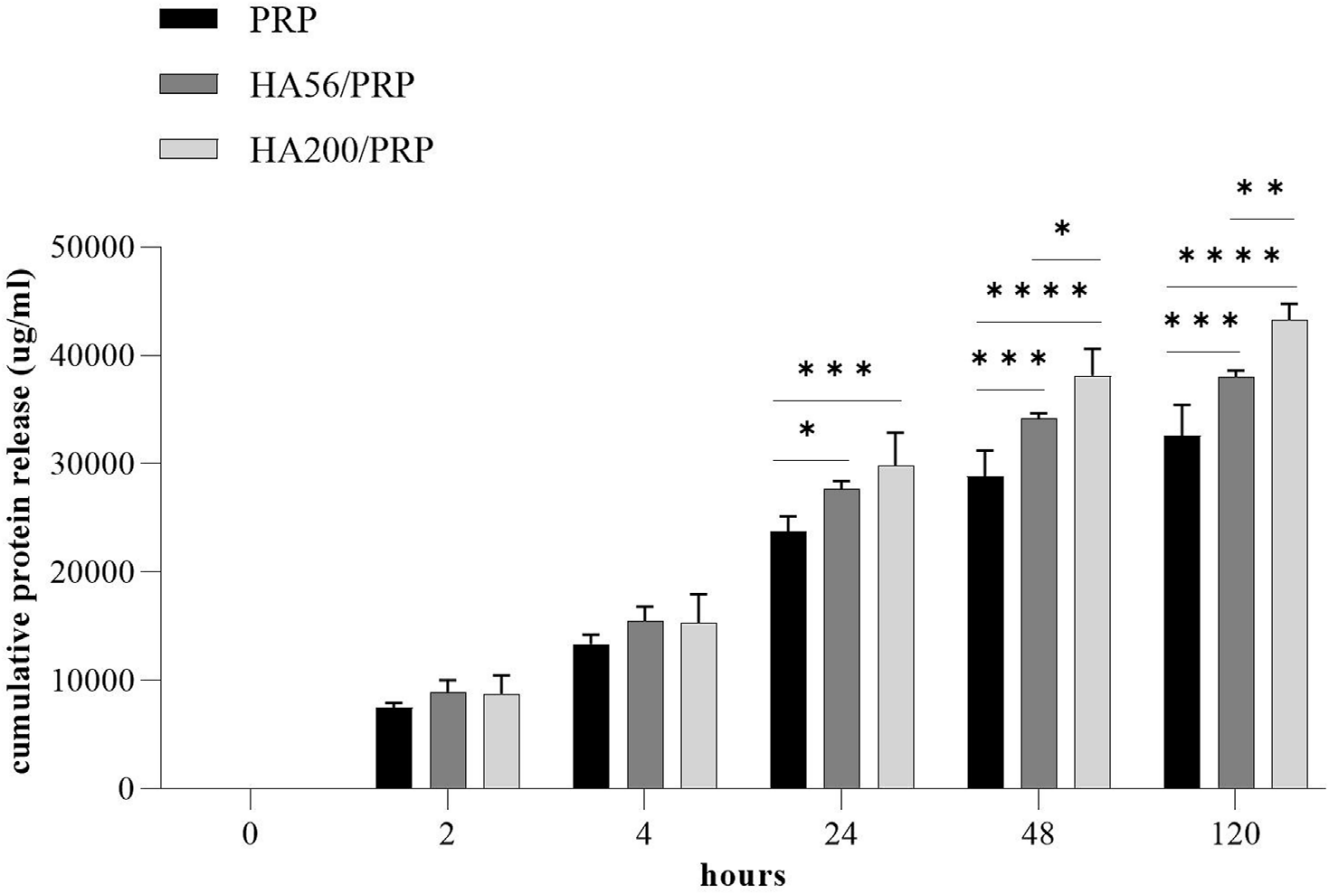
Protein release analysis. The total protein content was determined using the Bradford assay in the incubation media collected at different times from PRP or HA/PRP gel preparation. The experiments were performed in duplicate on three different gels for formulation (n = 3; results are expressed as mean ± SD).

**FIGURE 4:**
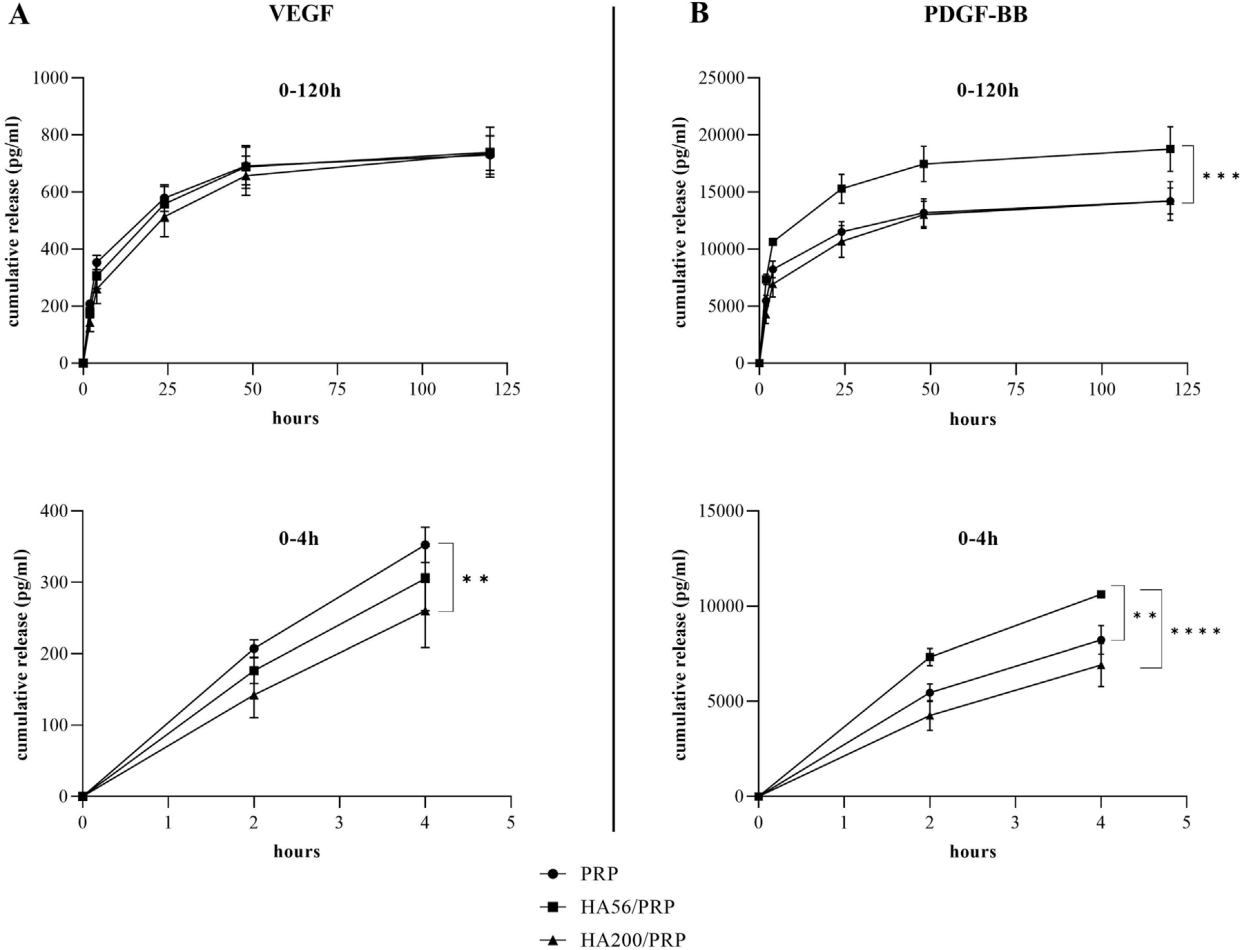
Growth factor release analysis. The amount of VEGF **(A)** and PDGF-BB **(B)** released in the incubation medium at different time points was determined, by ELISA assay, from PRP and HA/PRP formulations used immediately after their preparation and lyophilization. The curves represent the absolute values of the cumulative release (n = 3 for VEGF; n = 2 for PDGF-BB; results are expressed as mean ± SD).

Figure 3 shows the total protein release following the incubation of the different gel formulations with physiological solutions at 37°C and 5% CO_2_ and at different time points. Both HA200/PRP and HA56/PRP preparations released a slightly but significantly higher total amount of proteins than PRP at 24 h and in the subsequent points. The HA200/PRP preparation proved more effective than HA56/PRP in terms of protein release. PDGF-BB and VEGF release were evaluated in the incubation medium using the ELISA assay (Figure 4) to monitor PRP growth factor release over time. Interestingly, it was observed that HA56/PRP released a higher amount of PDGF-BB than PRP alone over time, while HA200/PRP also showed a slower release at early time points (0–4 h). A different protein profile was observed for the VEGF, where initially (0–4 h), the HA200/PRP formulation showed a statistically significantly lower growth factor release.

### 3.5 Lyophilized formulation stability: biological effect on cell proliferation

The biological activity of the freeze-dried products upon storage (0, 3, and 6 months) at 4°C and 25°C was assessed using the cell proliferation assay.

**FIGURE 5:**
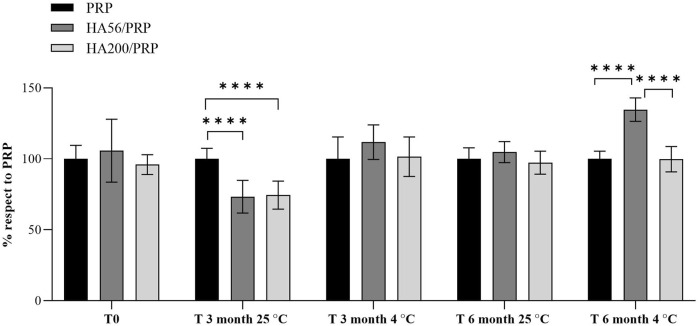
Proliferation rate of hFB cultured with PRP or HA/PRP formulations after their storage at 25°C and 4°C from the initial preparation time (T0) to 3 and 6 months (T3 and T6). Proliferation in the different preparations was calculated as a percentage relative to the PRP culture condition, which was set equal to 100%.

PRP and HA/PRP formulations induced a significantly higher proliferation rate of skin fibroblasts than the standard culture condition (CTR, 10% FCS) (data not shown). HA/PRP compositions used immediately after their preparation (t0) promoted cell proliferation comparably to PRP alone, as shown in Figure 5. After 3 months of storage at 4°C, the HA56/PRP sample was superior to PRP alone and HA200/PRP in inducing cell proliferation. This behavior was strongly accentuated, becoming statistically significant after 6 months, especially at 4°C. On the contrary, the preparations stored at 25°C did not offer an advantage to HA56/PRP and HA200/PRP over PRP alone. Nevertheless, it is evident that after 3 months of storage at this temperature, cells proliferate significantly better with PRP alone than HA formulations (Figure 5).

### 3.6 Wound healing and time-lapse video microscopy

**FIGURE 6:**
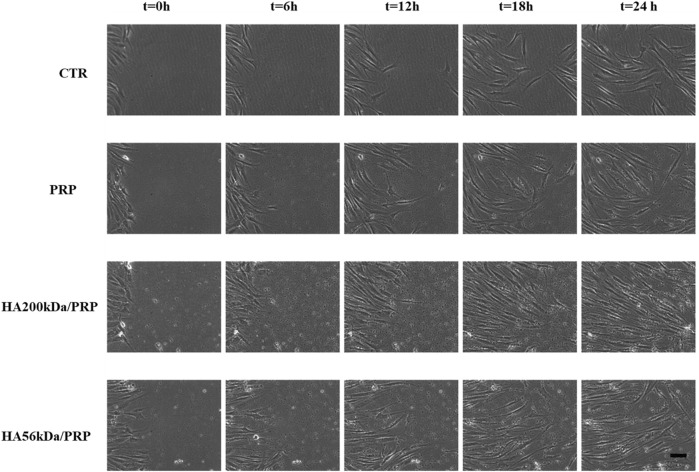
Representative micrograph images of hFB scratch assays immediately after the scratches and during the experiments. Scale bar: 100 µm.

All samples improved healing after 13 h, with HA/PRP samples proving more effective than PRP in accelerating repair, regardless of the HA molecular weight (200 and 56 kDa) (Figure 6). In the presence of HA, wound closure exceeded 80% within 24 h, whereas PRP-treated scratches alone reached approximately 60% repair. Statistical analyses reported in Table 3 (ANOVA test) revealed that HA200/PRP was the most effective treatment during the early stage of repair (12 h), whereas HA56/PRP successively accelerated the wound healing process more than other treatments.

**TABLE 3:** Wound closure at 12 and 24 h and statistical results of quantitative analysis of wound closure vs. time in the CTR (FCS) and in the presence of PRP and PRP/HA.

Wound closure		
Sample	t_12h_	t_24h_
CTR	20.8 ± 9.9	65.9 ± 7.8
PRP	31.8 ± 15.5	76.9 ± 11.9
HA200/PRP	38.1 ± 12.0*	91.5 ± 10.4**
HA56/PRP	33.7 ± 12.1	97.9 ± 6.5**#

*p < 0.05 vs CTR (12 h); **p < 0.005 vs. CTR (24 h); #*p* < 0.05 vs. PRP (24 h).

### 3.7 Gene and protein expression in hFB cells

**FIGURE 7:**
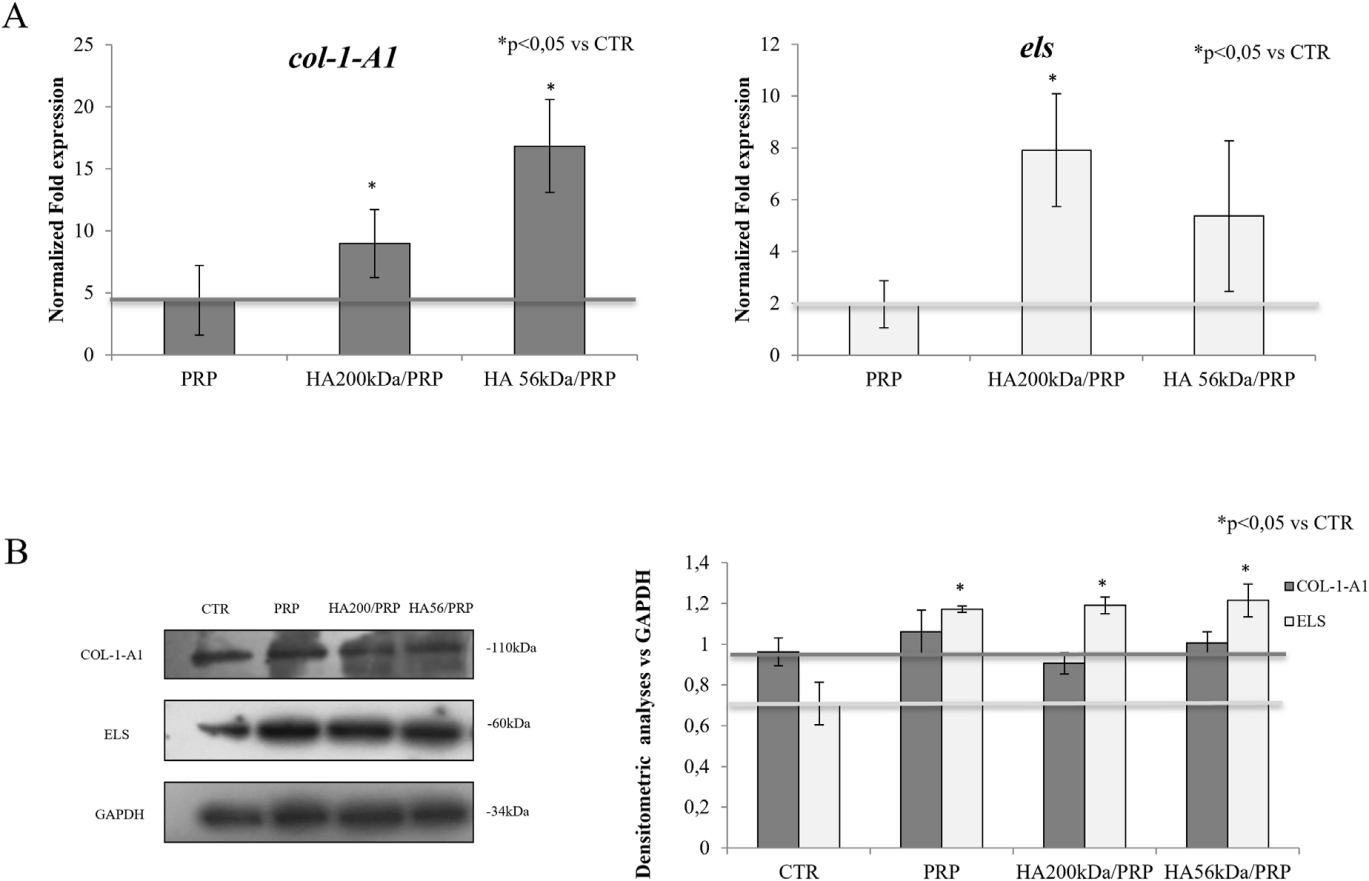
**(A)** RNA was extracted from hFB cells after 6 h of treatment, and qRT-PCR was performed for *COL1A1* and *ELS* gene expressions. **(B)** COL1A1 and ELS protein expressions, after 24 h, using Western blotting in hFB cells. The level of each protein was normalized to GAPDH expression, which was used as the housekeeping internal control.

ELS and COL1A1 expressions were investigated using real-time PCR and Western blotting. The results (Figure 7A) revealed, regarding gene expression, the capability of all the treatments to upregulate remodeling biomarkers. In particular, the presence of HA200 in the treatments significantly increased *COL1A1* and *ELS* expressions compared to the treatment with PRP alone. The addition of HA56, on the other hand, statistically upregulated only *COL1A1* gene expression. The Western blot reported in Figure 7B confirmed that the addition of HA in the PRP gels increased ELS expression, even if the modulation was less remarkable with respect to that found in the gene expression. Western blot experiments did not reveal COL1A1 modulations in the presence of HA, possibly because it is highly expressed even in the absence of treatments (CTR).

### 3.8 Lyophilized formulation stability: hFB scratch test (bioactivity upon storage)

**FIGURE 8:**
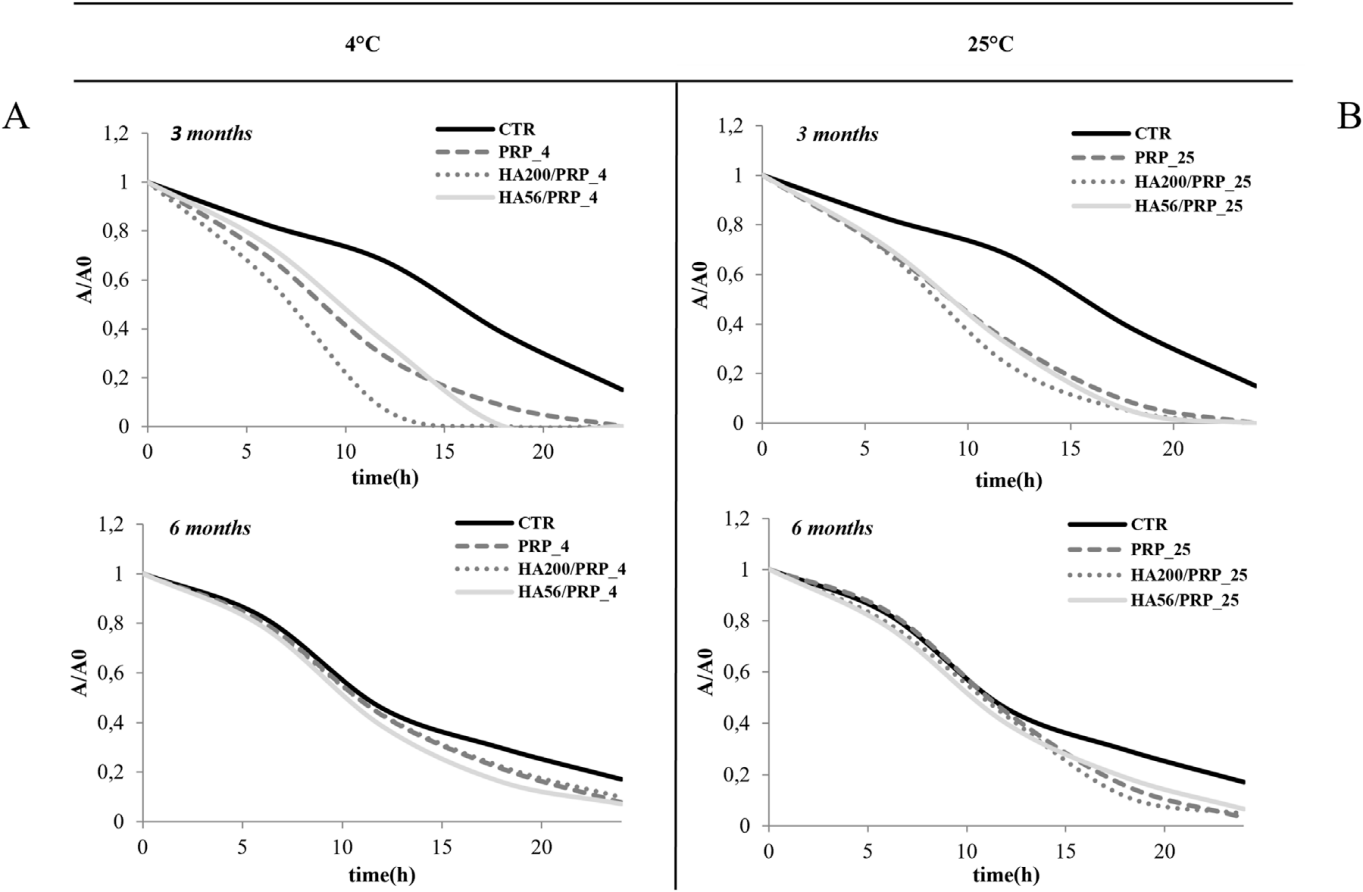
Stability evaluation using the TLVM scratch test **(A, B)**. Line graphs illustrate the area reduction (A/A0) of different samples over time at 4°C and 25°C, labeled (**A, B**). Each graph shows results for three and six months, comparing CTR, with PRP, HA200/PRP, and HA56/PRP.

The bioactivity of the lyophilized PRP and HA/PRP was also investigated during long-term storage at 4°C and 25°C (until 6 months). Experiments were performed in time-lapse using a scratch assay to determine whether HA could be effective not only in prompting tissue repair but also in protecting PRP from its natural tendency to lose its efficacy. Previous studies showed that PRP remained stable when lyophilized at −20°C, while information on storage in the refrigerator or at room temperature was not available. In view of the potential applications of this gel, the storage requirements (e.g., temperature and light) and shelf life are very important/valuable. At 3 months, the results (Figure 8) showed an improvement in scratch repair in the presence of different formulations, especially when PRP was combined with HA compared to the control (CTR, 1% FBS). These results were similar at both temperatures. At 4°C, HA200 kDa/PRP increased cell migration compared to HA56 kDa/PRP. At 25°C, after 6 months of storage, these differences between the treatments and the control diminished, suggesting that this duration is too long for PRP formulation storage, especially without refrigerating them. Table 4 shows that, at 12 h, healing relative to CTR exhibited more than a 1.5-fold increase in repair rate when PRP was combined with HA at both temperatures and up to 3 months. At 6 months, the variation between the treatments and the control diminished, but the statistical difference was evident vs. CTR, as reported in Table 4. The statistical analysis of these data is reported in Table 5.

**TABLE 4:** Comparison of healing at crucial time (12 h) at different times (3 and 6 months) and temperatures (4°C and 25°C).

Healing at 12 h compared to CTR (4°C)			
Sample	t_0_	3 months	6 months
PRP/CTR	1.45 ± 0.48	1.85 ± 0.32	1.06 ± 0.15
(HA200/PRP)/CTR	1.83 ± 0.58	2.62 ± 0.17	1.02 ± 0.19
(HA56/PRP)/CTR	1.62 ± 0.58	1.81 ± 0.18	1.18 ± 0.15

Healing at 12 h compared to CTR (25°C)			
Sample	t_0_	3 months	6 months
PRP/CTR	1.45 ± 0.48	1.55 ± 0.33	1.07 ± 0.17
(HA200/PRP)/CTR	1.83 ± 0.57	2.11 ± 0.51	1.03 ± 0.19
(HA56/PRP)/CTR	1.62 ± 0.58	1.83 ± 0.29	1.18 ± 0.24

**TABLE 5:** Statistical analyses of wound healing at crucial time (12 h) at 3 and 6 months.

Statistical analysis of healing at 12 h				
	4°C		25°C	
3 months	*p* < 0.05		*p* < 0.005	
	PRP	vs*. CTR*	HA56/PRP	vs*. CTR* vs*. PRP*
	HA56/PRP	vs*. CTR* vs*. HA200/PRP*		
			HA200/PRP	vs*. CTR* vs*. PRP*
	HA200/PRP	vs*. CTR* vs*. PRP*		
6 months	*p* < 0.005		*No significance*	
	HA56/PRP	vs*. CTR* vs*. PRP*		
	*p* < 0.05			
	HA200/PRP	vs*. HA56/PRP*		

## 4 Discussion

Scientific interest in developing new topical formulations and medical devices for wound healing has substantially increased in the recent years. In addition to accidental burnings or minor wounds that can occur from childhood to adulthood, there are also people affected by immune system diseases, metabolic disorders, and especially diabetes (diabetic foot ulcers), showing impaired healing and hampered tissue homeostasis re-establishment. In such cases, wound management is very difficult, leading to high health costs and poor outcomes. PRP has been proposed for many medical applications due to the high concentration of bioactive molecules and growth factors. It induces migration, proliferation, and biosynthetic activity of dermal fibroblasts, promoting extracellular matrix restoration and the differentiation of human dermal fibroblasts into myofibroblasts (Oneto and Etulain, 2021). We have previously set up a standardized protocol to obtain consistent results from different freeze-dried PRP preparations regarding the growth factor content and the biological activity (Muraglia et al., 2015). A pool of buffy coats and successive lyophilization strongly reduces the variability existing in the use of single units and also guarantees superior stability of the product. For clinical applications, PRP preparation and administration must be performed in specialized areas, possibly in a surgical room, using PRP derived from the patient’s own peripheral blood. These complex procedures may then be avoided by using allogeneic-derived PRP. In particular, patients who need treatment could take advantage of previously obtained preparations of enriched/stabilized and well-characterized PRP, with the reliability of the protocols and the safety constraints connected to blood processing in the hospitals (under the supervision of the regulatory offices EMA, FDA, etc.). To limit the issues related to PRP transport, stability, and storage, lyophilization is among the better approaches to be considered, and it is also frequently used to preserve cells and biomolecules in pharmaceutical and biomedical fields. During lyophilization, human proteins and growth factors need to be preserved using additives, which are added for specific applications of the resulting products. Considering the extensive experience in the study and characterization of hyaluronan (HA) families, which are also applied in wound management, HA was selected to play a dual role as a cryopreservant and a bioactive component. In particular, HA-based solutions were mixed with concentrated PRP, and gentle and prolonged shaking allowed the formation of entanglements between hyaluronic acid chains and proteins, growth factors, and chemokines present in the PRP preparations. Platelets were then embedded in viscous solutions containing a certain concentration of hyaluronan with well-known biochemical and biomechanical features. The obtained bioactive formulations were lyophilized under sterile conditions. The latter were either used immediately after freeze-drying or upon storage to evaluate their biological/biochemical functionality on human fibroblasts and establish their shelf life. The powders obtained presented no sticky grains or aggregates, and HA-based formulations proved more hygroscopic than PRP alone, as expected. SEM micrographs showed smooth coverage of platelets by HA and generally a diverse morphology for PRP and HA/PRP formulations. Total protein release studies showed that, within 24 h, HA/PRP preparations released a greater protein amount than PRP alone. Rao et al. (2022) recently reported a higher release of PDGF-BB from high-MW HA/PRP formulations (Rao et al., 2022). In our study, the PDGF-BB release was higher for HA56/PRP formulations, while PRP alone or HA200/PRP showed a similar behavior. The specific release of VEGF showed, at the beginning, a slower release for HA200/PRP preparations, while no differences among the three formulations could be highlighted at longer time points. These results are in line with the outcome of previous studies, which reported that HA or alginate-based gels modulate growth factor release from PRP (Kohei et al., 2016; Nardini et al., 2020). In particular, Spanò et al. (2018) reported different kinetics of release from PRP-based bioactive membranes, depending on the specific growth factor. Considering the rheological studies, we observed that HA56/PRP gels were less viscous than PRP, which, in turn, showed lower viscosity than HA200/PRP. This observation, together with the differences in molecular weight, may explain the release behavior reported in this study. Interestingly, all the formulations prompted healing after lyophilization; however, storage conditions affected the bioactivity. As expected, refrigerated samples better preserved their bioactivity, consistent with the nature of the formulations. However, in all experiments, HA/PRP preparations appeared to maintain functionality slightly longer than PRP alone, similarly to what was reported by Rao et al. (2022). The paper reports higher migration and proliferation for HA/PRP-treated samples, in which HA ranged from 1,400 to 800 kDa and was combined with PRP in a ratio of 3:1. All the formulations obtained within the present work, at each experimental time, even during storage, prompted wound healing, except for those stored for 6 months at the higher temperature. The shelf life in our case is longer than that reported before (Rao et al., 2022). Prompting wound closure is of great importance for the final medical/physiological outcome since the faster the repair, the lower the risk of infections (chronic wound) (Falanga et al., 2022). In this respect, the viscosity and adhesiveness of the formulation are important for achieving prolonged persistence in the wound bed/site, ensuring slow and sustained release of growth factors, possibly optimizing their therapeutic effect. In our experiments, both HA and PRP appeared to have the greatest impact on accelerating wound repair, presumably because the polymer better retains the bioactive molecules through entanglements with proteins, resulting in a modulated release and an improved performance. It is known that HA200 is among the most used hyaluronans in biomedical applications (Frenkel, 2014). On the other hand, the positive effect of HA56, despite its lower viscosity, could be useful for maximizing cell migration and obtaining a more concentrated HA solution while remaining applicable and manageable. As reported in the results, HA56 appeared to be more permissive to the growth factor release, probably due to the lower extent of entanglements. It is also interesting to note that some key biomarkers in tissue remodeling and skin homeostasis, like collagen 1A1 and elastin, were prompted by our treatments, suggesting that the human fibroblast molecular machinery is strongly activated toward repair (Samadi et al., 2019; Guszczyn et al., 2017; Abuaf et al., 2016). However, gene expression data did not directly reflect the protein quantification using Western blotting. Nevertheless, a modulation was evidenced for elastin, while a less significant improvement was found for collagen 1A1. These two molecules, which are responsible for the structure and function of the extracellular matrix, together with glycosaminoglycans, should be present in the correct physiological ratio to ensure tissue texture, elasticity, and the continuity of the repaired area with healthy tissue (D'Agostino et al., 2017; Chowdhury et al., 2021). Regarding stability during storage, lyophilization of the mixture maintained the efficacy of the formulations for up to 3 months, longer than previously reported, while the presence of HA improved fibroblast migration, consistent with previous reports. The positive results presented in this study foster further studies to improve the persistence and efficacy of the proposed HA/PRP mixtures on the wound bed *in vivo*. The main limitation of the presented study is related to the simplified *in vitro* model based on scratched fibroblasts. *In vivo* diffusion of the growth factors that are released into the wound bed may directly experience a harsh microenvironment that could not be fully resembled *in vitro* in our experiments. Nevertheless, comparative analyses of novel diverse formulations need a consolidated and reproducible experimental model to obtain robust outcomes.

## 5 Conclusion

Novel gels based on low-molecular-weight HA and PRP were investigated in this work to determine whether HA could positively influence the already well-known beneficial properties of PRP in wound repair. Results showed that both before and right after lyophilization, the formulas improved fibroblast proliferation and wound repair in an *in vitro* model. The addition of HA improved the efficacy of PRP; however, the two different HA sizes proposed in this study showed similar behavior, despite a slight difference in viscosity. Accelerated wound repair is important in wound management as it helps reduce the risk of infection at the injury site, which is a major factor in the development of chronic wounds. The new formulations developed showed beneficial effects in tissue reparation and matrix remodeling, suggesting their potential positive role in wound care. Both hyaluronic acid and PRP have been shown, when used separately, to support many cellular processes, especially in tissue regeneration. However, the mechanisms of action are diverse, like the biophysical properties; thus, their combination, in addition to providing a slightly superior stability during storage, permitted the slow release/better availability of the bioactive components of PRP to immediately feed cells in the wound bed. Low-molecular-weight hyaluronan has been demonstrated as the most powerful in prompting skin tissue repair. Overall, our *in vitro* experiments suggest that the new combined lyophilized formulations could be beneficial in wound management, offering a ready-to-use solution HA/PRP without the need for on-site preparation and displaying a functional and favorable role in tissue healing.

## Data Availability

The raw data supporting the conclusions of this article will be made available by the authors, without undue reservation.
