# Supplementary figures and images for: Novel platelet-rich plasma/ hyaluronic acid lyophilized formulations for wound healing applications
Source: Front Bioeng Biotechnol. 2025 Sep 10;13:1619633. doi: 10.3389/fbioe.2025.1619633 (PMC12457828; doi:10.3389/fbioe.2025.1619633)

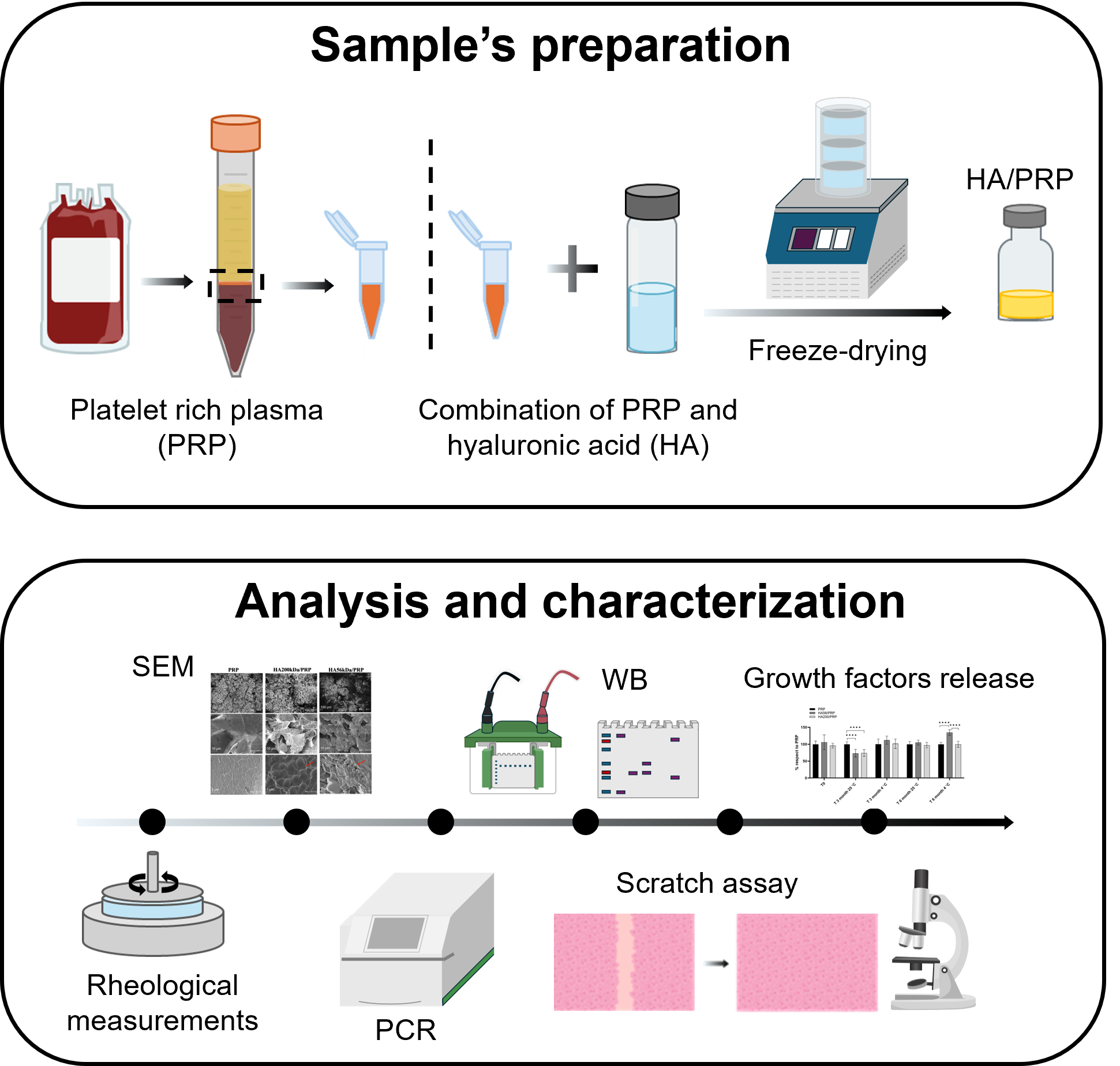

Supplement: Supplementary file 1 [file Image2.tif]

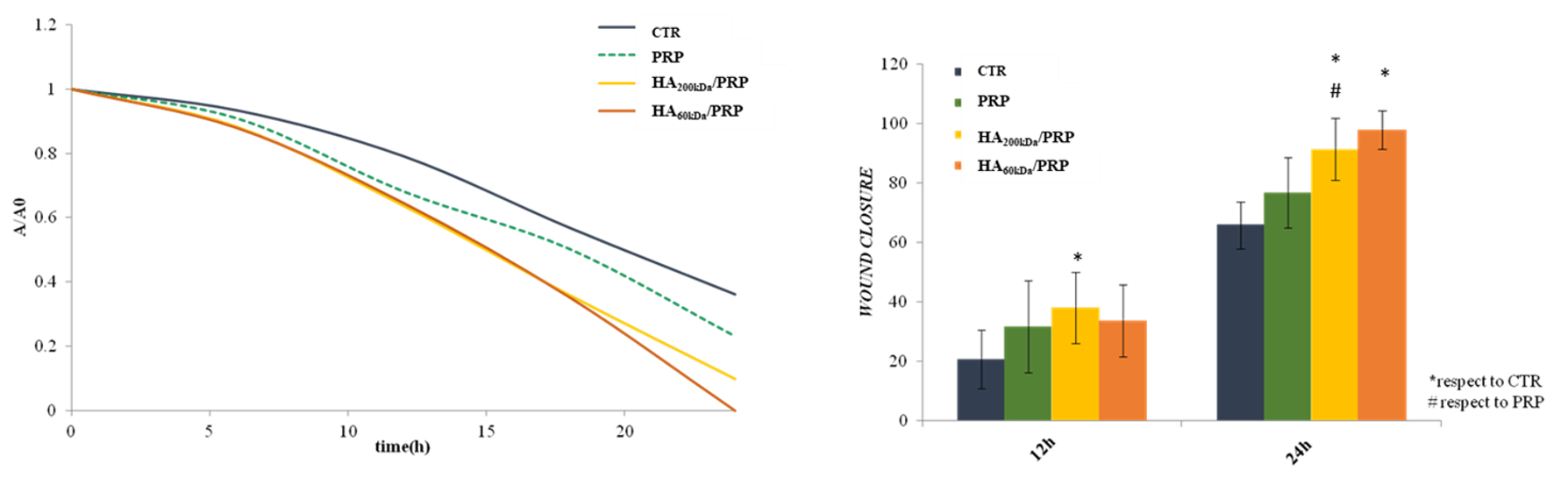

Supplement: Supplementary file 2 [file Image1.tif]
